# Relationships Between Sports Club Participation and Health Determinants in Adolescents and Young Adults

**DOI:** 10.3389/fspor.2022.918716

**Published:** 2022-06-15

**Authors:** Alexis Barbry, Annie Carton, Hervé Ovigneur, Jérémy Coquart

**Affiliations:** ^1^Univ. Lille, Univ. Artois, Univ. Littoral Côte d'Opale, ULR 7369 - URePSSS - Unité de Recherche Pluridisciplinaire Sport Santé Société, Lille, France; ^2^Université de Rouen-Normandie, Centre des Transformations des Activités Physiques et Sportives, Rouen, France; ^3^L'Institut des Rencontres de la Forme, Wattignies, France; ^4^Univ. Artois, Univ. Lille, Univ. Littoral Côte d'Opale, ULR 7369 - URePSSS - Unité de Recherche Pluridisciplinaire Sport Santé Société, Liévin, France

**Keywords:** physical activity, body weight, gender, adolescence, BMI class

## Abstract

Physical fitness is a powerful marker of health in adolescents and young adults. The purpose of this study was to measure the relationships between age, sex, body mass index, and sports club participation on physical fitness. The population included 49,988 participants (23,721 girls and 26,267 boys) who were divided into five age groups (11–12, 13–14, 15–16, 17–18, and 19–21 years). Body mass index was calculated. Physical fitness was assessed with the Diagnoform^®^ Tonic battery. Sports club participation was also documented. The practiced sport was collected. The effects of age, sex, body mass index class, and sports club participation were tested. Boys' PF increased with age at a faster rate and was better than that of girls, except for flexibility (*p* < 0.001). For girls, a decrease was observed in endurance, speed and flexibility at 17-18 years. Sports club participation was greater for boys at every age. Obese participants had the lowest physical fitness and sports club participation. Sports club participation increased physical fitness. Team sports seemed best for improving physical fitness, except flexibility. The study shows that sports club participation may be a key element for building health in adolescents. Preventive healthcare projects that promote sports club are needed to target sports club dropouts (obese adolescents and girls). Bridges should be built between physical education classes and sports clubs in adolescence to improve the health status of young people.

## Introduction

Body composition and physical fitness (PF), which includes cardiorespiratory endurance, coordination, muscular strength, speed and flexibility, are powerful markers of health in adolescents and young adults (Ortega et al., 2008).

PF increases as adolescents grow older (Tambalis et al., 2016), but overall boys seem to have better PF than girls during adolescence (Tambalis et al., 2016; Tomkinson et al., 2018). For example, the maximal oxygen uptake ($\overset{∙}{V}{\text{O}}_{2}$max expressed in ml.kg^−1^.min^−1^), which is an indicator of cardiorespiratory endurance, is known to slightly decrease during girls' growth and to stagnate or slightly increase during boy's growth (Wilmore et al., 2017). Regarding the other PF components, coordination, power and speed generally improve (Barnett et al., 2016; Tambalis et al., 2016) with age, but the improvements are greater in boys (Tomkinson et al., 2018). Flexibility seems to improve during adolescence in both sexes with a slower increase after 15–16 years (Lee et al., 2017), but girls seem to be more flexible than boys during adolescence (Tambalis et al., 2016; Tomkinson et al., 2018). Therefore, it seems important to consider age and sex when measuring overall PF and its components (i.e., cardiorespiratory endurance, coordination, muscular strength, speed, and flexibility).

Although a stabilizing trend has been noted worldwide in adolescents (Olds et al., 2011), the prevalence of overweight and obesity remains at particularly high levels. These high levels of prevalence can be explained by the increase in sedentary behaviors and the decrease in physical activities (PA) in adolescents (Booth et al., 2019). Low sports club participation (SCP) may also be a factor. Indeed, given that overweight and obesity have been negatively linked with health and PF (Lopes et al., 2019), SCP might protect against the development of overweight and obesity and poor PF. Notably, adolescents (regardless of corpulence) with SCP show on average 20–27% better PF compared to those without SCP (Telford et al., 2016). However, the links between the type of sport practiced, overweight/obesity and PF are not well known.

The main aim of the current study was therefore to evaluate the relationships between age, sex, body mass index (BMI) class and SCP (in 5 sports) on PF in young girls and boys between 11 and 21 years old. We assumed that (i) PF would increase with age in both sexes, (ii) boys would have higher PF than girls, (iii) normal-weight subjects would be those with the best PF, (iv) SCP would be more frequent in boys and normal-weight participants, and (v) some sports (rather than others) would be better for preventing overweight and obesity and/or improving PF.

## Materials and Methods

### Experimental Approach

This retrospective study was based on data from the “Observatory of French physical fitness^®^” using the physical tests included in the Diagnoform Tonic^®^ battery, which was previously validated (Mouraby et al., 2012). Data were collected in 13 regions of France (mostly in cities) between 2011 and 2019 in schools and universities. All data obtained for the “Observatory of French physical fitness^®^” were anonymized, declared, and approved by the National Commission on Informatics and Liberty (n° 1232206). The aims were carefully explained to the study participants. Written informed consent was obtained from all volunteers and their parents prior to study commencement in accordance with international ethical standards and the Declaration of Helsinki. This study was approved by the National Ethics Committee for Research in Sports Sciences Institutional Review Board 00012476-2021-28-05-109. Data were recorded in an electronic data system. They were categorized in five age groups: 11–12 years (11.4 ± 0.5), 13–14 years (13.5 ± 0.5), 15–16 years (15.3 ± 0.5), 17–18 years (17.4 ± 0.5), and 19–21 years (19.6 ± 0.8). These age groups were chosen because they represent grade levels in schools and universities in France.

### Individual Characteristics

Height and body mass were self-reported by the participants. BMI was calculated. The corpulence was assessed using the International Obesity Task Force scale (Cole et al., 2000). This scale was created from a large database of children and adolescents (i.e., from birth to 18 years old) from six different countries. Centiles curves have been created with the BMI to create specific cut off points according to age and sex (e.g., for more information, see Cole et al.). Moreover, information on SCP and the sport practiced were collected. Before the Diagnoform^®^ Tonic tests, the sports educator asked the participants the following question: “Are you a member of a sports club?”. If the answer was yes, the sports educator asked them: “What sport do you practice the most?” Only one sport can be given to the instructor. The sport most represented in each of the four categories of the Official Bulletin of French Physical Education (Ministère de l'Education Nationale et de la Jeunesse., 2019) was separately identified for girls and boys. As recommended and suggested by Barbry et al. (2021), we identified one sport rather than a group of sports. These four categories were: C1 for sports where the goal is to achieve a maximal physical performance measurable on a given day (e.g., athletics, swimming, cycling…), C2 for sports where the goal is to move by adapting movements in a varied and uncertain environment (e.g., rock climbing, horseback riding, sailing…), C3 for sports where the goal is to achieve a physical performance that is artistic or acrobatic (e.g., dance, gymnastics, figure skating…), and C4 for sports where the goal is to lead and manage an individual or collective confrontation (e.g., soccer, basketball, tennis…).

### Tests of the Diagnoform^®^ Tonic

The validity and reliability of the tests included in the Diagnoform Tonic^®^ to evaluate the PF components have been previously shown (Mouraby et al., 2012) and recently used by Duclos et al. (2021). The tests were supervised by a sports educator and administered in the following order:

#### Cardiorespiratory Endurance

Cardiorespiratory endurance was assessed by a 3-min shuttle run. The aim was to cover the maximum distance (in meters) during a 3-min run by running back and forth with the obligation to put one foot on a line at each changeover. This test was validated against the 20-m shuttle run reference test (Mouraby et al., 2012).

#### Coordination

**Figure 1:**
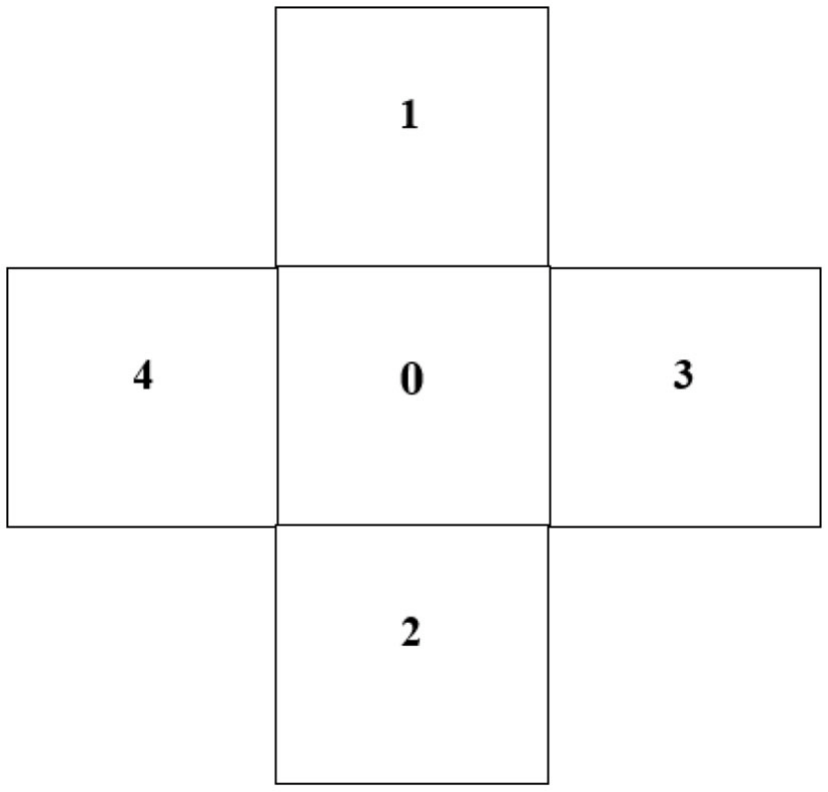
Representation of the cross test.

Coordination was assessed by the cross test (Figure 1). The cross is composed of five numbers, each in a square with 50-cm sides (0 in the center square, 1 above the center, 2 below the center, 3 to the right of center, and 4 to the left). The participants were asked to jump with feet together as quickly as possible into the squares of the cross for 30 s. They had to do so following the strict number order while jumping into the central square (square 0) between each square. The feet had to be within the square separation lines. Each jump on a number (except 0) earned 1 point if the order was respected. Each successful cycle earned 4 points. If a mistake was made, the participants had to start a new cycle. This test was previously used by Mouraby et al. (2012).

#### Power

Lower limb power was assessed by the standing broad jump test. From a starting position immediately behind a line, standing with the feet approximately shoulder-width apart, participants jumped as far as possible with their feet together. The result was recorded in cm. A nonslip hard surface was used to perform the test. This test was validated by Fernandez-Santos et al. (2015).

#### Speed

Speed was measured from a 30-m run performed as fast as possible (in seconds). The participants stood still in a comfortable position, feet behind the starting line, with no rocking movement. The test began on a whistle and was concluded when they crossed the finish line. The performance was recorded to the nearest tenth of a second. This test was validated by Vicente-Rodríguez et al. (2011).

#### Arm Strength

Muscular strength for the upper limbs was assessed by the bent-knee push-up test. Participants lay on the ground with hands on both sides of the shoulders. They lifted the feet but kept the knees on the ground. The back was straight and the hips were maintained. They descended slowly by bending the elbows so that the humerus and radius made a right-angle corner (90°). They then had to rise to the initial position by using arm strength. They were asked to repeat this movement as many times as they could. The result was the maximum number of push-up repetitions completed. A supervisor monitored to ensure that the position was correct and counted the number of repetitions.

#### Flexibility

Flexibility was assessed by assessing the participant's ability to bend forward from a standing position and touch the floor. From a standing position and with both legs straight, the participants progressively flexed their trunk and reached down as far as possible with their hands. They maintained the position for 3 s. The test results were indexed as follows: a score of 5 for placing the hands entirely flat on the ground, 4 for three fingers on each hand touching the ground, 3 for fingers reaching the ankle (on the talus), 2 for fingers reaching the tibia (midway between the astragal and the tibial tuberosity), and 1 for fingers/hands reaching the knees (patella). This field test was validated by Perret et al. (2001).

### Statistical Analysis

Data are expressed as the mean ± standard deviation for continuous variables. For categorical data, the sample size and frequencies are presented. For continuous variables, the normality of the distribution was verified with a Kolmogorov-Smirnov test, and equality of variances was analyzed with Levene's test.

For the continuous variables, a one-way ANOVA (analysis of variance) was executed to test the effect of age class for each sex (girls and boys), with the age (11–12 vs. 13–14 vs. 15–16 vs. 17–18 vs. 19–21 years) conditions as the between-subject factors. When significant differences were obtained, a Bonferroni *post-hoc* test was conducted to determine where the differences lay. For categorical data, the frequencies were compared using Pearson's chi-square test.

To test the effects of BMI class (underweight vs. normal weight vs. overweight vs. obesity), SCP and practiced sport on the performances during the PF tests, one-way ANOVAs were executed. A Bonferroni *post-hoc* test was conducted when significant differences were noted. Last, the proportion of participants in each BMI class by sport was analyzed with Pearson's chi-square test.

Statistical significance was set at *p* < 0.05 and all analyses were performed with the Statistical Package for the Social Sciences (release 18.0, Chicago, IL, USA).

## Results

### Age and Sex

For boys, significant differences were found for endurance between each age group (*p* < 0.001, Table 1) in contrast to girls, for whom no significant difference was found between 13–14 and 15–16 years. Girls in the 11–12 age group had better endurance than girls in the 17–18 group (*p* = 0.043, Table 1). Regardless of group, boys always had better endurance than girls (*p* < 0.001).

**Table 1:** Socio-demographic characteristics, sport licenses and performances according to age and gender.

	**Girls**					**Boys**				
	**11–12 years**	**13–14 years**	**15–16 years**	**17–18 years**	**19–21 years**	**11–12 years**	**13–14 years**	**15–16 years**	**17–18 years**	**19–21 years**
	**7403 (14.8%)**	**3685 (7.4%)**	**9422 (18.8%)**	**1955 (3.9%)**	**1256 (2.5%)**	**8011 (16.0%)**	**4049 (8.1%)**	**9643 (19.3%)**	**2745 (5.5%)**	**1819 (3.6%)**
Sport	5183 (70.0%)^b, c, d, e, f^	2292 (62.2%)^a, c, d, f^	5434 (57.7%)^a, b, d, f^	1032 (52.8%)^a, b, c, e, f^	755 (60.1%)^a, d, f^	6172 (77.0%)^b, c, d, e, f^	2908 (71.8%)^a, e, f^	6781 (70.3%)^a, e, f^	1930 (70.3%)^a, e, f^	1544 (84.9%)^a, b, c, d, f^
licenses
(n)
Body	42.4 ± 9.7^b, c, d, e, f^	51.7 ± 9.9^a, c, d, e, f^	54.6 ± 8.9^a, b, d, e, f^	56.9 ± 9.8^a, b, c, e, f^	58.7 ± 9.3^a, b, c, d, f^	41.8 ± 9.5^b, c, d, e, f^	54.9 ± 12.0^a, c, d, e, f^	62.7 ± 11.4^a, b, d, e, f^	68.1 ± 11.0^a, b, c, e, f^	71.1 ± 10.3^a, b, c, d, f^
mass
(kg)
Height	152 ± 8^b, c, d, e, f^	161 ± 7^a, c, d, e, f^	164 ± 6^a, b, e, f^	164 ± 7^a, b, e, f^	165 ± 6^a, b, c, d, f^	151 ± 8^b, c, d, e, f^	166 ± 10^a, c, d, e, f^	174 ± 7^a, b, d, e, f^	177 ± 7^a, b, c, e, f^	178 ± 7^a, b, c, d, f^
(cm)
BMI	18.3 ± 3.3^b, c, d, e^	19.8 ± 3.4^a, c, d, e^	20.4 ± 3.0^a, b, d, e, f^	21.3 ± 3.5^a, b, c, f^	21.6 ± 3.1^a, b, c, f^	18.3 ± 3.2^b, c, d, e^	19.9 ± 3.4^a, c, d, e^	20.7 ± 3.2^a, b, d, e, f^	21.8 ± 3.1^a, b, c, e, f^	22.4 ± 2.7^a, b, c, d, f^
(kg.m^−2^)
*Underweight*	301 (4.1%)^b, c, d, e^	99 (2.7%)^a, d, e^	267 (2.8%)^a, d, e^	145 (7.4%)^a, b, c, e, f^	137 (10.9%)^a, b, c, d, f^	280 (3.5%)^c^	134 (3.3%)	270(2.8%)^a, d, e^	108 (3.9%)^c, f^	70 (3.8%)^c, f^
*(n Normal*	5952 (80.4%)^b, c^	3082 (83.6%)^a, c, e, f^	8299 (88.1%)^a, b, d, e, f^	1603 (82.0%)^c, e^	989 (78.7%)^b, c, d, f^	6411 (80.0%)^b, c, d, e^	3160 (78.0%)^a, c, d, e, f^	8025 (83.2%)^a, b, f^	2251 (82.0%)^a, b^	1506 (82.8%)^a, b, f^
weighted
(n)
*Overweight*	953 (12.9%)^b, c, d, e^	409 (11.1%)^a, c, d, e, f^	709 (7.5%)^a, b, f^	158 (8.1%)^a, b, f^	96 (7.6%)^a, b, f^	1063 (13.3%)^b, c, d^	605 (14.9%)^a, c, d, e, f^	1097 (11.4%)^a, b, f^	324 (11.8%)^a, b, f^	213 (11.7%)^b, f^
(n)
*Obesity*	197 (2.7%)^c, f^	95 (2.6%)^c, f^	147 (1.6%)^a, b, d, e, f^	49 (2.5%)^a, c^	34 (2.7%)^c, f^	257 (3.2%)^c, d, f, e^	150 (3.7%)^c, d, e, f^	251 (2.6%)^a, b, e, f^	62 (2.3%)^a, b^	30 (2.1%)^a, b, c, f^
(n)
Endurance	505 ± 64^b, c, d, e, f^	511 ± 74^a, d, e, f^	510 ± 75^a, d, e, f^	500 ± 89^a, b, c, e, f^	524 ± 79^a, b, c, d, f^	538 ± 71^b, c, d, e, f^	571 ± 81^a, c, d, e, f^	605 ± 76^a, b, d, e, f^	623 ± 81^a, b, c, e, f^	646 ± 71^a, b, c, d, f^
(m)
Coordination	26.9 ± 7.8^b, c, d, e, f^	27.9 ± 8.9^a, c, d, e^	29.4 ± 8.3^a, b, e, f^	29.4 ± 9.1^a, b, e, f^	32.6 ± 8.9^a, b, c, d, f^	26.5 ± 8.3^b, c, d, e, f^	27.8 ± 9.3^a, c, d, e^	30.5 ± 9.2^a, b, d, e, f^	32.5 ± 9.8^a, b, c, e, f^	36.3 ± 9.2^a, b, c, d, f^
Power	139 ± 27^b, c, d, e, f^	146 ± 29^a, c, d, e, f^	150 ± 30^a, b, d, e, f^	152 ± 33^a, b, c, e, f^	160 ± 29^a, b, c, d, f^	153 ± 28^b, c, d, e, f^	176 ± 33^a, c, d, e, f^	195 ± 32^a, b, d, e, f^	209 ± 34^a, b, c, e, f^	220 ± 28^a, b, c, d, f^
Speed (s)	6.03 ± 0.75^b, c, d, e, f^	5.67 ± 0.80^a, c, d, e, f^	5.42 ± 0.78^a, b, d, e, f^	5.52 ± 0.83^a, b, c, e, f^	5.32 ± 0.77^a, b, c, d, f^	5.74 ± 0.72^b, c, d, e, f^	5.19 ± 0.80^a, c, d, e, f^	4.71 ± 0.73^a, b, d, e, f^	4.58 ± 0.62^a, b, c, e, f^	4.31 ± 0.64^a, b, c, d, f^
(cm)
Arm	20.5 ± 14.3^b, c, d, e, f^	21.4 ± 13.0^a, d, e, f^	21.2 ± 13.1^a, d, e, f^	23.0 ± 15.5^a, b, c, e, f^	29.1 ± 16.7^a, b, c, d, f^	30.1 ± 18.1^b, c, d, e, f^	35.5 ± 18.9^a, c, d, e, f^	41.6 ± 20.0^a, b, d, e, f^	47.1 ± 23.2^a, b, c, e, f^	55.2 ± 23.2^a, b, c, d, f^
strength
(n)
Flexibility	3.80 ± 0.91^b, c, d, e, f^	3.96 ± 0.89^a, c, f^	4.05 ± 0.90^a, b, d, e, f^	3.97 ± 0.92^a, c, f^	4.04 ± 0.94^a, c, f^	3.25 ± 0.88^b, c, d, e, f^	3.41 ± 0.91^a, c, d, e, f^	3.54 ± 0.90^a, b, d, e, f^	3.61 ± 0.92^a, b, c, e, f^	3.75 ± 0.93^a, b, c, d, f^
(score/5)

*Legend: BMI, body mass index.*

*^a^Significantly different from 11–12 years (p <0.05).*

*^b^Significantly different from 13–14 years (p <0.05).*

*^c^Significantly different from 15–16 years (p <0.05).*

*^d^Significantly different from 17–18 years (p <0.05).*

*^e^Significantly different from 19–21 years (p <0.05).*

*^f^Significantly different between girls and boys (p <0.05)*.

Regarding coordination, a significant increase was found between all age groups (*p* < 0.001, Table 1) for both sexes. Coordination was significantly higher in the 11- to 12-year-old girls compared to their male counterparts (*p* = 0.001). On the other hand, for the 15–16 age group and older, boys had higher coordination than girls (*p* < 0.001).

For both boys and girls, significant differences were found between each age group regarding power (*p* < 0.05, Table 1) and speed (*p* < 0.001). For both PF components, boys were significantly better than girls (regardless of age group, *p* < 0.001).

For both sexes, arm strength increased significantly with age (*p* < 0.05, Table 1) except for girls in the 13–14 group, whose arm strength was not significantly different from that of the 15–16 group. Arm strength was significantly higher in boys than girls (regardless of age group, *p* < 0.001).

For flexibility, significant differences were found between all age groups for boys and girls (*p* < 0.01, Table 1), except between the 13–14 group and the two highest age groups for girls (*p* > 0.05), and between the 17–18 and 19–21 age groups (*p* = 0.296). Flexibility was significantly higher in girls than boys (regardless of age group, *p* < 0.001).

### BMI Class

Endurance, coordination, power, and speed were significantly higher in normal-weight participants, followed in descending order by those who were underweight, then overweight, and then obese (*p* < 0.001, Table 2).

**Table 2:** Performances during the tests of physical fitness according to class of body mass index.

	**Underweight**	**Normal weighted**	**Overweight**	**Obesity**
Endurance (m)	543 ± 82^b, c, d^	556 ± 84^a, c, d^	514 ± 89^a, b, d^	460 ± 89^a, b, c^
Coordination	28.3 ± 8.5^b, c, d^	29.5 ± 9.0^a, c, d^	26.8 ± 8.7^a, b, d^	24.2 ± 8.1^a, b, c^
Power (cm)	163 ± 33^b, c, d^	168 ± 38^a, c, d^	153 ± 39^a, b, d^	135 ± 37^a, b, c^
Speed (s)	5.40 ± 0.80^b, c, d^	5.27 ± 0.86^a, c, d^	5.65 ± 1.00^a, b, d^	6.15 ± 1.07^a, b, c^
Arm strength (n)	27.6 ± 17.2^b, d^	31.6 ± 20.3^a, c, d^	26.3 ± 19.1^b, d^	20.4 ± 14.1^a, b, c^
Flexibility (score/5)	3.51 ± 0.97^b, c^	3.71 ± 0.94^a, c, d^	3.62 ± 0.95^a, b^	3.55 ± 0.91^b^

*^a^Significantly different from underweight (p <0.05).*

*^b^Significantly different from normal weighted (p <0.05).*

*^c^Significantly different from overweight (p <0.05).*

*^d^Significantly different from obesity (p <0.05)*.

The normal-weight participants had greater arm strength than the other groups (*p* < 0.001, Table 2). They were also significantly more flexible than their underweight, overweight and obese counterparts (*p* < 0.001, Table 2). There were significantly more underweight, overweight and obese individuals among those without sports club membership (*p* < 0.001, **Table 5**).

### SCP

For girls, SCP decreased at 13–14 compared to their counterparts aged 11–12 (*p* < 0.001, Table 1), 13–14 and 15–16 (*p* < 0.001, Table 1), and 15–16 and 17–18 (*p* < 0.001, Table 1). However, it increased between 17–18 and 19–21 (*p* < 0.001, Table 1). For boys, SCP also decreased between 11–12 and 13–14 (*p* < 0.001, Table 1) and also increased between 17–18 and 19–21 (*p* < 0.001, Table 1). At each age, boys were more often in a sports club than girls (*p* < 0.001, Table 1).

The performances of participants without sports club membership were always lower than that of participants with membership, regardless of the PF component (*p* < 0.001, Table 3).

**Table 3:** Performances during the tests of physical fitness according to sport license.

	**Sport license**	**Non-sport license**
	**(*n* = 34,031)**	**(*n* = 15,957)**
Endurance (m)	563 ± 84^a^	516 ± 86^a^
Coordination	30.1 ± 9.1^a^	26.8 ± 8.3^a^
Power (cm)	171 ± 38^a^	154 ± 37^a^
Speed (s)	5.22 ± 0.87^a^	5.59 ± 0.93^a^
Arm strength (n)	33.7 ± 20.1^a^	23.8 ± 16.2^a^
Flexibility (score/5)	3.74 ± 0.94^a^	3.59 ± 0.94^a^

*^a^Significantly different from between the participants with sport licence and non-sport license (p <0.05)*.

Girls and boys in a soccer or basketball club had significantly better endurance than those in other sports clubs (*p* < 0.001, Table 4). Dancers (and boys riding horses) had the lowest endurance (*p* < 0.05).

**Table 4:** Performances during the tests of physical fitness according to sport in girls and boys.

**Girls**	**Dance (*n* = 3,465)**	**Horseback riding (*n* = 1,633)**	**Swimming (*n* = 946)**	**Soccer (*n* = 538)**	**Basket-ball (*n* = 986)**
Endurance (m)	508 ± 64^b, c, e, f^	516 ± 69^a, d, e^	520 ± 63^a, d, e^	539 ± 67^a, b, c^	540 ± 73^a, b, c^
Coordination	29.2 ± 8.3^b, d, e^	28.1 ± 8.2^a, d, e^	28.9 ± 8.0^d, e^	30.3 ± 9.7^a, b, c^	31.0 ± 9.2^a, b, c^
Power (cm)	145 ± 26^d, e^	147 ± 26^d, e^	147 ± 29^d, e^	161 ± 31^a, b, c, e^	156 ± 30^a, b, c, d^
Speed (s)	5.67 ± 0.75^b, d, e^	5.54 ± 0.72^a, c, d, e^	5.67 ± 0.79^b, d, e^	5.30 ± 0.76^a, b, c^	5.37 ± 0.73^a, b, c^
Arm strength (n)	21.0 ± 12.9^c, d, e^	21.1 ± 12.8^c, d, e^	26.4 ± 17.9^a, b^	25.6 ± 15.5^a, b^	24.7 ± 14.5^a, b^
Flexibility (score/5)	4.20 ± 0.86^b, c, d, e^	3.90 ± 0.87^a, c^	4.02 ± 0.88^a, b, d, e^	3.82 ± 0.88^a, c^	3.84 ± 0.89^a, c^
**Boys**	**Dance (*****n*** **=** **237)**	**Horseback riding (*****n*** **=** **1,243)**	**Swimming (*****n*** **=** **739)**	**Soccer (*****n*** **=** **6,270)**	**Basket-ball (*****n*** **=** **1,504)**
Endurance (m)	561 ± 80^c, d, e^	558 ± 93^c, d, e^	580 ± 78^a, b, d, e^	604 ± 79^a, b, c^	603 ± 79^a, b, c^
Coordination	30.8 ± 9.8^b^	27.5 ± 8.9^a, d, e^	29.2 ± 8.9^d, e^	30.3 ± 9.4^b, c, e^	31.2 ± 9.7^b, c, d^
Power (cm)	180 ± 42^e^	180 ± 37^e^	178 ± 39^d, e^	185 ± 36^c, e^	191 ± 37^a, b, c, d^
Speed (s)	5.22 ± 0.79^d, e^	5.15 ± 0.68^d^	5.22 ± 0.93^d, e^	4.92 ± 0.84^a, b, c^	4.94 ± 0.84^a, c^
Arm strength (n)	36.2 ± 20.6^c, e^	32.2 ± 17.5^c, d, e^	40.6 ± 25.5^a, b^	39.8 ± 19.8^b^	40.8 ± 20.0^a, b^
Flexibility (score/5)	3.76 ± 0.92^c, d, e^	3.49 ± 0.80	3.52 ± 0.91^a^	3.47 ± 0.89^a^	3.42 ± 0.92^a^

*^a^Significantly different from dance (p <0.05).*

*^b^Significantly different from horseback riding (p <0.05).*

*^c^Significantly different from swimming (p <0.05).*

*^d^Significantly different from soccer (p <0.05).*

*^e^Significantly different from basketball (p <0.05)*.

Coordination was significantly higher in girls playing the two team sports (soccer and basketball, Table 4), but for boys it was significantly higher only for those playing basketball (*p* < 0.05, Table 4).

Girls playing soccer had significantly more power than other girls (*p* < 0.05, Table 4), whereas boys playing basketball were significantly more powerful than other boys (*p* < 0.05, Table 4). Soccer was the sport where the highest speed was observed, for both girls and boys.

In girls, significantly greater arm strength was found in soccer players, basketball players and swimmers (*p* < 0.05, Table 4). In boys, the greatest arms strength was found in basketball players and swimmers (Table 4).

Dancers had significantly higher flexibility than athletes in all other sports (*p* < 0.001, Table 4), with the exception of boys riding horses (*p* = 0.071, Table 4).

Moreover, there were significantly more underweight and normal-weight individuals among dancers and riders compared to those in other sports (*p* < 0.05, Table 5). Inversely, significantly fewer dancers and horseback riders were overweight (*p* < 0.001). Obesity was significantly more prevalent among basketball players compared to dancers (*p* = 0.014), horseback riders (*p* = 0.024), and soccer players (*p* = 0.024).

**Table 5:** Prevalence in each class of body mass index according to sports.

	**Underweight**	**Normal weighted**	**Overweight**	**Obesity**
Non-sport license (*n* = 15,957)	695 (4.4%)^f^	12,690 (79.5%)^f^	1,980 (12.4%)^f^	592 (3.7%)^f^
Sport license (*n* = 34,031)	1,116 (3.3%)^f^	28,588 (84.0%)^f^	3,647 (10.7%)^f^	680 (2.0%)^f^
Dance (*n* = 3,702)	156 (4.2%)^c, d, e^	3,184 (86.0%)^c, d, e^	305 (8.2%)^c, d, e^	57 (1.5%)^e^
Horseback riding (*n* = 1,757)	77 (4.4%)^c, d, e^	1,527 (86.9%)^c, d, e^	128 (7.3%)^c, d, e^	25 (1.4%)^e^
Swimming (*n* = 1,685)	49 (2.9%)^a, b^	1,418 (84.2%)^a, b^	187 (11.1%)^a, b^	31 (1.8%)
Soccer (*n* = 6,808)	195 (2.9%)^a, b^	5,752 (84.5%)^a, b^	746 (11.0%)^a, b^	115 (1.7%)^e^
Basket-ball (*n* = 2,490)	72 (2.9%)^a, b^	2,085 (83.7%)^a, b^	273 (11.0%)^a, b^	60 (2.4%)^a, b, d^

*^a^Significantly different from dance (p <0.05).*

*^b^Significantly different from horseback riding (p <0.05).*

*^c^Significantly different from swimming (p <0.05).*

*^d^Significantly different from soccer (p <0.05).*

*^e^Significantly different from basketball (p <0.05).*

*^f^Significantly different between non-sport license and sport license (p <0.05)*.

## Discussion

The aim of the study was to measure the influence of age, sex, corpulence and SCP on PF in girls and boys between 11 and 21 years old. Differences in the evolution of PF components with age were observed (Table 1). Cardiorespiratory endurance increased with age in boys, who always had higher cardiorespiratory endurance than girls (Table 1). For boys, this increase could be explained by enhanced running economy with growth (Ratel, 2018) despite the stagnation of $\overset{∙}{V}{\text{O}}_{2}$max expressed in relative value (Wilmore et al., 2017). For girls, this result might be explained by the decline in $\overset{∙}{V}{\text{O}}_{2}$max expressed in relative value with growth (Wilmore et al., 2017), possibly due to the increase in percent body fat and the smaller increase in muscular mass (kg) compared to boys after 14 years (Malina et al., 2004). This sex difference might also be explained by the progressive increase with age in the hematocrit level for boys, which implies a greater capacity to transport oxygen (Van Praagh, 2007). Our results for coordination suggested an increase with age for both sexes (Table 1). Barnett et al. (2016) reported similar results and suggested the explanation might be the opportunities offered to adolescents in schools to practice PA that develop motor skills. However, our results also showed a greater increase in coordination in boys compared to girls after 15 years. The slower increase in girls might be due to lower engagement in PA (Jaakkola and Washington, 2012). According to Tomkinson et al. (2018), the anaerobic PF components (i.e., power, speed, and arm strength) globally increased with age (Table 1). Increases in body size, changes in body composition and neuromuscular maturation are the main hypotheses (Malina et al., 2004). For example, fat-free mass is a key determinant of anaerobic performance (Vardar et al., 2007) and is known to increase with age, especially in boys (Malina et al., 2004), and this would explain the better results compared to girls. Flexibility increased with age in boys but tended to decrease in girls after 16 years before increasing again at 19–21 years. Lee et al. (2017) found similar results with an increase in flexibility with age in both sexes but a slight progression observed for girls after 15 years. According to other author (Ratel, 2018), the drop that we observed after 16 years could be explained by lengthening of the diaphysis, which always precedes muscular lengthening, causing muscular stiffness. Our results also showed that girls always had better flexibility than boys, regardless of age. The difference in tissue density (which was less for girls) might help them to be more flexible (Beunen and Malina, 1988; Malina et al., 2004). Furthermore, girls practiced more sports (i.e., gymnastics and dance) (Ministère des Sports., 2002) that specifically develop flexibility during training sessions.

In line with the findings of other studies (Mak et al., 2010; Lopes et al., 2019), our results suggested that the obese participants had the lowest PF and the normal-weight participants had the higher performances for each PF component (Table 2). A couple of explanations should be noted. First, the obese participants had to carry their excess body mass, which increased their energy cost and therefore reduced their performances (Deforche et al., 2003). Second, they were less engaged in PA than their normal-weight counterparts, and PA is known to improve PF (Raistenskis et al., 2016). A further interesting finding was that the participants who were not in a sports club were more often overweight or obese (Table 5). An earlier study pointed out that SCP is a way to combat obesity (Telford et al., 2016), but obese individuals are generally not interested in this type of participation, possibly because training sessions are not adapted to their needs. Indeed, sports clubs for adolescents and young adults tend to focus on competition and not on the playful and leisure sides of sports.

Our results showed that in each age group, boys were more often involved in SCP than girls (Table 1). The girls might have thought that they had less potential for sports activity than boys (Vigneron, 2006) and thus they preferred to invest in other leisure activities. In line with another study (Ministère des Sports., 2002), we also found that the older the teenagers were, the less they practiced in sports clubs, although with a gender difference (Table 1). Indeed, the difference expressed in percentage between boys and girls also increased with age (10% difference at 11–12 years vs. 35% at 17–18 years) (Table 1). In general, the sports dropout rate is higher in girls than boys during adolescence, and some adolescents have indicated that a lack of time, loss of pleasure, and even too much homework would explain this outcome (Ministère des Sports., 2002). Moreover, the sports that are most abandoned during adolescence are artistic sports like gymnastics and dance (Ministère des Sports., 2002), which are more frequently practiced by girls.

In line with other authors (Telford et al., 2016), we found that the participants with SCP had higher PF than the others (Table 3). Our study went further by comparing PF for those sports most practiced in each of the categories of the Official Bulletin of French Physical Education (Ministère de l'Education Nationale et de la Jeunesse., 2019) (Table 4). Participants of both sexes who practiced a team sport (i.e., soccer or basketball) had the best cardiorespiratory endurance, whereas dancers and horseback riders had the lowest endurance. These results are not surprising because soccer and basketball are essentially composed of intermittent aerobic effort, which positively impacts cardiorespiratory endurance (Ben Abdelkrim et al., 2007; Fernandes et al., 2015). In contrast, dance and riding horses are less demanding for the cardiorespiratory system. The boys and girls who played basketball had higher coordination. The cross test, which was used to evaluate coordination, also requires numerous changes in direction, which occur often in basketball. Moreover, basketball is well known to develop motor coordination (DiFiori et al., 2018). The athletes in team sports also had higher power and speed, which is unsurprising given that these PF components are determinants of performance in team sports (Ardern et al., 2015). Furthermore, the test used for speed may have been an advantage for the soccer players because in the other selected sports running for 30 m at an extremely high intensity is rare. For arm strength, boys and girls who practiced swimming, and basketball for boys, had higher performances, which could be explained by the importance of muscular strength in these sports (Aspenes et al., 2009; Kariyawasam et al., 2019). Indeed, Aspenes et al. (2009) found that muscular strength is correlated with swim velocity, and for basketball, arm strength is required in various movements like shooting (Kariyawasam et al., 2019). Dancers had higher flexibility. Indeed, flexibility is a major determinant of performance for dancers, as without good flexibility movements are restrained. We can assume that the dancers worked on it during training sessions.

Our study also suggested that dancers and horseback riders were more often underweight and normal-weight and thus less often overweight and obese than the other athletes. For dance, this result can be explained by the dancer's morphology, which is generally thinner with less body fat and a lower BMI (Edita et al., 2005). On the other hand, horseback riders often have weight restrictions because the horse's speed is a key determinant of performance (Jeon et al., 2018). Notably, basketball players were more often obese than the participants in other sports. Silva et al. (2013) found that adolescent basketball players had the greatest BMI compared to players of other team sports, which might be explained by their greater muscle mass.

Our study had both strengths and limitations. The main strength was the large sample of participants (*n* = 49,988). Concerning the study limitations, we did not know the number of sports practiced by the participants. The participants only had to indicate whether they were in a sports club and, in that case, the sport they practiced the most. We also did not know the practice frequency or level. Another limitation was the percentage of participation in the different schools and universities. Indeed, a higher percentage of high school students did not give their consent to carry out the tests (about 30–40%) compared to middle school students (about 15–20%). We can assume that those who did not participate were those who disliked sports and PA or who had difficulties with them (overweight and obese participants). Finally, despite our large amount of data, we are aware that we are not perfectly representative of all French regions. Other studies could look at the relationships between SCP and the same health determinants as ours in some specific territories (e.g., in the countryside or in priority neighbourhoods).

## Conclusion

Our results suggest that PF generally increased at a faster rate for boys, who had better PF except in flexibility. For girls, cardiorespiratory endurance slowly increased until 15–16 years and decreased at 17–18 years. Girls had higher coordination until 15 years old. Decreases in speed and flexibility were also observed at 17–18 years for girls. The prevalence of overweight and obesity was higher for boys than girls. The normal-weight participants had the best PF and the obese participants had the worst. Boys were more often in a sports club than girls. As the participants grew older, they engaged less in these clubs. The sports club participants had better PF and were more often normal-weight, whereas those not in clubs were more often overweight or obese. Team sports seemed to be the best way to develop cardiorespiratory endurance, coordination, power and speed. For arm strength, both team sports and swimming seemed to be the best. Dance seemed to be the best to develop flexibility. The promotion of SCP is essential for French youth because it may be a way to combat the burden of overweight and obesity and increase health. Adolescents who do not practice in sports clubs may become more physiologically frail adults. Future research could be conducted to understand more precisely the way to engage the dropouts of SCP in a sports club. Research could be realized to explain the high level of PF of adolescents playing team sports compared to adolescents practiced other sports. This original research should be of interest to public health, education and sport policies to jointly reflect on a federal sports offer that is more fun and not just competitive as a continuation of physical education classes in order to improve the health of young French people. Finally, it could be interesting to develop a network between the city (e.g., sports and health departments), the local sports club and the physical education teachers to work together and build bridges for promoting physical activity for adolescents and young adults.

## Data Availability Statement

The raw data supporting the conclusions of this article will be made available by the authors, without undue reservation.

## Ethics Statement

The current study was reviewed and approved by the Institutional Review Board of 00012476-2021-28-05-109. Written informed consent to participate in this study was provided by the participants' legal guardian/next of kin.

## Author Contributions

AB, AC, HO, and JC contributed to the study design, literature review, data gathering, manuscript writing, and data analyses and interpretation. All authors commented on the draft and contributed to the final version, approved the publication of the manuscript, and agreed to be accountable for all aspects of the work.

## Funding

This publication has been supported by Lille University in the context of its open science support policy.

## Conflict of Interest

The authors declare that the research was conducted in the absence of any commercial or financial relationships that could be construed as a potential conflict of interest.

## Publisher's Note

All claims expressed in this article are solely those of the authors and do not necessarily represent those of their affiliated organizations, or those of the publisher, the editors and the reviewers. Any product that may be evaluated in this article, or claim that may be made by its manufacturer, is not guaranteed or endorsed by the publisher.
